# Service Providers Perspectives on Personal Recovery from Severe Mental Illness in Cape Town, South Africa: A Qualitative Study

**DOI:** 10.1007/s10597-021-00904-8

**Published:** 2021-10-20

**Authors:** Fadia Gamieldien, Roshan Galvaan, Bronwyn Myers, Katherine Sorsdahl

**Affiliations:** 1 Department of Psychiatry and Mental Health, Alan J. Flisher Centre for Public Mental Health, University of Cape Town, 46 Sawkins Road, Rondebosch, Cape Town, 7700 South Africa; 2 Division of Occupational Therapy, Department of Health and Rehabilitation Sciences, University of Cape Town, Cape Town, South Africa; 3 Faculty of Health Sciences, Curtin enAble Institute, Curtin University, Perth, WA Australia; 4 Alcohol, Tobacco and Other Drug Research Unit, South African Medical Research Council, Cape Town, South Africa; 5 Division of Addiction Psychiatry, Department of Psychiatry and Mental Health, University of Cape Town, Cape Town, South Africa

**Keywords:** Recovery, Severe mental illness, Low-and-middle-income countries, Service providers

## Abstract

Severe mental illnesses (SMI) contribute significantly to the global burden of disease. In low-and-middle-income countries (LMICs), the treatment gap impacts the clinical and personal recovery of people living with an SMI. The drive to reduce this treatment gap in LMICs makes it pertinent to understand service providers’ views on recovery from SMI. Semi-structured interviews and focus groups with service providers from health services and non-profit organisations in the Western Cape Province, South Africa, were conducted in this qualitative study. Seventeen participants were purposively selected, and data were thematically analysed. Three major themes emerged: delineating recovery, available services supporting recovery from SMI, and facilitators and barriers to recovery at the service level. Health services favoured clinical over personal recovery. Participants thought that many service users’ personal recovery from SMI was hindered by intersecting social, economic, cultural, and political inequalities that extended beyond the influence of the health sector.

## Introduction

Severe mental illnesses (SMI) (schizophrenia spectrum disorders, non-organic psychotic disorders, and bipolar affective disorder) are some of the main causes of years lived with disability (YLDs) (Vos et al., 2017). Despite schizophrenia being a low prevalence disorder, the Global Burden of Disease study (2016) ranked it as the 12th most disabling disorder out of 310 injuries and diseases (Vos et al., 2017), contributing 1.7% of total YLDs globally, equivalent to 13.4 million YLDs (Charlson et al., 2018). The burden associated with these disorders is four times greater in low-and-middle-income countries (LMICs) than high-income (HICs) due to the substantial treatment gap (Charlson et al., 2018). Additionally, people living with SMI require complex interventions to address the social and economic difficulties they face (Charlson et al., 2018).

In many LMICs, available treatment for SMI is primarily biomedical and provided at specialised psychiatric facilities on an inpatient or outpatient basis. However, as many LMICs allocate insufficient resources to SMI services in primary levels of care treatment for SMI remains inadequate in these settings (Iseselo & Ambikile, 2017), and there is little integration between hospitals and community-based services (Eaton et al., 2011). Services at primary levels of care are limited to the collection of medication at clinics but community based services which facilitate community integration are limited (Lund et al., 2010). Inadequate care for people with SMI holds negative consequences for the person with the SMI, family, and community. These consequences include human rights violations (Asher et al., 2017), high relapse rates (Lund et al., 2010), homelessness (Smartt et al., 2019), risks of restraint and assault (A. Fekadu et al., 2019a, 2019b), adverse intergenerational effects on family members (W. Fekadu et al., 2019a, 2019b), caregiver burden (Shibre et al., 2012), premature mortality (Teferra et al., 2011), poverty and food insecurity (Jenkins et al., 2010; Teferra et al., 2013), stigma and discrimination (Egbe et al., 2014), and economic strain on carers (Addo et al., 2018), and unemployment (Ebuenyi et al., 2018).

Inadequate care also impacts mental health service users’ (MHSUs) community integration and recovery (Aldersey et al., 2017). Biomedical approaches to SMI focus on symptom remission as the main indicator of (clinical) recovery. In contrast, personal recovery approaches emphasise functional improvement, social inclusion, community integration, illness management, access to employment and family support as crucial markers of recovery alongside symptom remission (Aldersey et al., 2017; Slade, 2009). While there is no universally applicable definition of personal recovery from SMI, there is agreement that it involves developing meaning and purpose while living with an SMI (Mathew et al., 2018; Slade, 2009).

There is growing interest in understanding the meaning of recovery from multiple stakeholder perspectives, not just from the perspectives of MHSUs as experts by experience (Swerdfager, 2016). Several studies have explored the views of service providers on recovery-oriented practice. A systematic review identified three major conceptualisations of recovery-oriented practice: clinical recovery, personal recovery and service-defined recovery (Le Boutillier et al., 2015). Service defined recovery is of interest to health system reform as the goals and financial needs of the organisation influence how recovery translates into practice (Le Boutillier et al., 2015). Of the 22 studies included in this review, only one study came from a LMIC. The interest in research on recovery from SMI in LMICs is relatively new. A scoping review exploring the recovery of people living with SMI in LMICs (Gamieldien et al., 2021) found similarities but also contextual differences in how stakeholders from high income countries and LMICs frame personal recovery from SMI.

There is a shortage of research on service provider perspectives of recovery from SMI in Africa. In Nigeria, a qualitative study conducted with 312 doctors across eight health institutions found that provider stigma was a barrier to recovery (Adewuya & Oguntade, 2007). A qualitative study conducted in Ethiopia revealed that healthcare workers favoured a biomedical approach to recovery while other stakeholders saw medication as only one care component (Mall et al., 2017). A qualitative study exploring social worker perspectives of recovery from SMI was conducted in Tshwane, South Africa revealed a lack of knowledge and skills around recovery-oriented practices, a biomedical focus on illness and deficits, and stereotypical attitudes towards mental illness, which influenced practice (Bila, 2019). Research indicates that the attitudes of healthcare providers (Egbe et al., 2014) impede recovery, and this needs to be better understood (Egbe et al., 2014). Yet perspectives of recovery from SMI from a multidisciplinary team perspective have rarely been reported. Addressing this gap is important as providers from different disciplines may have different views on illness and recovery. This paper aims to explore the diverse views of a range of healthcare providers in the public health and non- profit organisation (NPO) sectors on (i) their understanding of recovery, (ii) the services in the Western Cape that support recovery and (iii) the barriers and facilitators to recovery.

## Method

This paper adheres to the Consolidated Criteria for Reporting Qualitative Research (COREQ) (Tong et al., 2007). In this qualitative, descriptive study (Kim et al., 2017; Lambert & Lambert, 2012; Neergaard et al., 2009; Sandelowski, 2010), we used the framework method (Gale et al., 2013; Ritchie & Spencer, 2002) as it is a pragmatic approach to systematically analyse qualitative data. This study was executed by the first author (FG) and supervised by the co-authors (KS, RG and BM).

### Study Context

Cape Town is a densely populated city in the Western Cape Province, which includes a large area outside the city centre. This area is referred to as the Cape Flats. During apartheid, people designated as Black and Coloured (Erasmus, 2000) were forcibly removed from their homes and relocated to the Cape Flats which is characterised by high levels of violence, crime, and poverty (Bowers du Toit, 2014). Specialised mental health services in the Western Cape Province of South Africa are rendered from three psychiatric hospitals: Valkenberg, Lentegeur and Stikland Hospitals. These hospitals each cater for different parts of the Cape Flats. MHSUs with an SMI are often referred directly to these psychiatric hospitals for interventions (Lund et al., 2010). Once admitted, they are treated by multidisciplinary team (MDT) members. MHSUs are discharged from hospital to the outpatient service of the admitting tertiary hospital. After that, they are discharged and collect their psychotropic medication from their closest community health centre (CHC). In Cape Town, a few NPOs are offering community-based mental health services to MHSUs. These NPOs are located on or close to the premises of the psychiatric hospitals and not on the Cape Flats, where many MHSUs live. The NPO services provide supported living options, day programmes and in-hospital activities not included in the hospital rehabilitation programme. These NPO-run services are staffed by health professionals including social workers, registered counsellors, occupational therapists, auxiliary workers and MHSUs.

### Participants

All service providers and service managers in the public mental health and NPO sectors involved in the the provision of services for people with SMI were eligible for participation in the study. Service providers and service managers were identified using purposive sampling, and maximum variation was sought to ensure that stakeholders at different levels of service and emanating from a range of public health and NPO sectors were included. Participants included service managers, heads of institutions, multidisciplinary team members and community-based service providers. Participants trained as health professsionals included psychiatrists, occupational therapists, social workers, psychologists and nurses.. In total, 17 participants were recruited for the study.

### Procedure

The interviews and focus group discussions (FGDs) (Bradshaw et al., 2017) were conducted by the main researcher (FG), a female occupational therapist from the Cape Flats with experience conducting qualitative research in mental health. Potential participants were contacted via email, informing them of the study and inviting them to participate. A convenient time and location were identified to conduct the interviews and FGDs, which ran for one hour each. Eleven individual interviews were conducted, and participants were only interviewed once. Additionally, two FGDs were held for participants affiliated with the same organisation and unable to meet individually. One FGD had two participants, and the other FGD had four participants. This pragmatic approach to data collection allowed for flexibility in FGD to accommodate participants availability in their natural service provision settings (Bradshaw et al., 2017).This smaller group allowed for rich discussion between participants which was guided by FG (Gill et al., 2008; Morgan, 2012; Ritchie & Spencer, 2002). An interview guide was used (Bradshaw et al., 2017), and participants were invited to share their views on: (a) the mental health services they offer to MHSUs; (b) their understanding of clinical vs personal recovery; (c) available services for SMI; (d) accessibility of services; (e) caregiver inclusion in services; and (f) benefits and gaps in current services. All participants provided written informed consent before inclusion in the study. The interviews and FGDs were conducted in English, in a private room at the participants’ workplace. The discussions were audio-recorded and transcribed verbatim. FG made field notes during and after each interview and FGD. This study adheres to the tenets of the Declaration of Helsinki. Ethical approval for the study was provided by the University of Cape Town’s Faculty of Health Sciences Research Ethics Committee (HEC 655/2018) and the Western Cape Government Health Resarch Committe (WC_201902_010).

### Data Analysis

NVivo12 software aided data analysis. The framework approach allowed us to analyse the data in a systematic manner (Gale et al., 2013). The five key stages of framework analysis was followed, namely: (i) familiarisation with data, (ii) identifying a thematic framework, (iii) indexing, (iv) charting, and (v) mapping and interpretation of the data. The analytical framework consisted of codes and categories that were developed from the research question and its key concepts and this was used to sift, chart and sort the data (Ritchie & Spencer, 2002). The initial coding and identification of preliminary categories and themes was conducted by the first author (FG). After that, the first author and second author (KS) familiarised themselves with the data and together developed a thematic framework. FG and KS then coded the first five transcripts independently. The two authors met regularly to refine the codes and gain consensus to establish an intercoder agreement (Creswell & Poth, 2018). FG continued coding the remaining eight transcripts. Coding continued until no new information emerged from the transcripts. Once themes were identified, member checks were done, allowing participants to comment on the interpretation of the data (Nowell et al., 2017). Member checking did not yield new information. As a reflexive practice, FG kept a research journal to document her thoughts and views on personal recovery during the interview and coding process. To ensure trustworthiness, FG employed reflexivity, peer debriefing, and an audit trail to promote quality, authenticity, and truthfulness of findings.

## Results

The demographic characteristics of the study participants are described in Table 1. In total, 17 service providers agreed to participate in interviews and FGDs. The following health professionals were represented: occupational therapists (3); social workers (5); psychologists (2); psychiatrists (2); and nurses (2). Two participants were community rehabilitation workers, and one was a volunteer with a finance background. Of the 17 participants, nine were employed in the NPO sector, and the rest where employed in the public health sector. In terms of gender, three participants were male.

**Table 1:** Demographic characteristics of participants, n = 17

No	Participant ID	Gender	Profession	Sector
1	OT 1	Female	Occupational therapist	Health
2	OT 2	Female	Occupational therapist	NPO
3	OT 3	Female	Occupational therapist	NPO
4	SW 1	Female	Social worker	Health
5	SW 2	Female	Social worker	NPO
6	SW 3	Female	Social worker	NPO
7	SW 4	Female	Social worker	NPO
8	SW 5	Female	Social worker	NPO
9	P 1	Female	Psychologist	Health
10	P 2	Female	Psychologist	Academia
11	D 1	Male	Psychiatrist	Health
12	D 2	Male	Psychiatrist	Health
13	N 1	Female	Nurse	Health
14	N 2	Male	Nurse	Health
15	C 1	Female	Community rehabilitation worker	NPO
16	C 2	Female	Community rehabilitation worker	NPO
17	V 1	Female	Volunteer manager	NPO

Results are reported according to the three major themes identified in the framework analysis: (1) delineating recovery, (2) available services supporting recovery from SMI in the Western Cape, and (3) facilitators and barriers to personal recovery within services. Quotes are attributed to participants using the following abbreviations: social workers (SW); psychologists (P), psychiatrists (D), occupational therapists (OT), nurse (N), volunteer manager (V), and community rehabilitation worker (C).

## Delineating Recovery

When asked about the definition of recovery, the importance placed on personal and clinical recovery depended on the providers’ work setting. Service providers indicated that health services favoured clinical over personal recovery. According to several service providers (from different professions), focusing on clinical recovery allowed them to objectively measure MHSUs’ progress, especially while MHSUs were in the psychiatric hospital.

*The clinical things are given because this is a psychiatric hospital; it is the clinical model. It is a medical model. It is about medication and symptoms, that is almost like the baseline.* (SW3)

Clinical recovery was linked to MHSUs’ mental state and their behaviour: “if they adhere to their medication, take it on time and they actively participate in groups, maybe then they can recover*”*. According to service providers, clinical recovery starts in the hospital, where the focus is to give medication and intervene to alleviate MHSUs’ acute symptoms.

*To get a person clinically well has to be our priority as a [psychiatric] hospital. That is what we are here for. And if it is medication, then we must push that and support it because I mean, I believe in pot plants, and I believe in nice duvets, but I do not think that makes sense alone when you are hearing things, seeing things and feeling at your most disconnected as a human can ever feel.* (P1)

According to these providers, personal recovery followed clinical recovery and was viewed as an extension of clinical recovery that started with MHSUs understanding their diagnosis.

*And I think an integral part of that actual recovery starts with the client in terms of insight, in terms of intellectually and on a personal level having the insight into exactly what your diagnosis is about. How your diagnosis works, your chances for recovery and then coupled with internal motivation and all of those things.* (OT2)

All service providers expressed the sentiment captured by one service provider who said that the journey of personal recovery involves a “slow, complex and cyclical process”. Service providers associated personal recovery with the following: the ability to set goals, engage in self-care, exercise choice and autonomy, and access resources. While service providers believed in the importance of personal recovery, this concept was not embedded in the hospital’s services or communicated in the physical environment of the hospital.

*So, the other thing about recovery is accepting the complexity. It is realising that as important as your therapy or medication is, looking after what you eat, doing a bit of exercise and having friendships and relationships and working on something important to you. So, clinical recovery is seen as this endpoint with a whole lot of measurables. Recovery with a capital R is how I like to refer to personal recovery.* (D1)

*Your journey is a long journey, you understand, we cannot do it all here, but we start putting back the pieces. (N2)*

Engaging in the journey of personal recovery “is about finding meaning and purpose in your life, and that is an endless process.” Service providers described some indicators of personal recovery. MHSUs highlighted vital features such as “quality of life” and “being an active member of the community”. According to the service providers, indicators of recovery varied, and they acknowledged the reality of relapses along the way:

*So recovery for me is probably equal to someone having a good quality of life, maintaining a sense of commitment towards sobriety, and being an active member of society. Certainly, the need to have a family, to be loved, and to be independent is still something that they view as significant for them. They do recover, which means that they will, at a certain point, experience symptoms again. (D2)*

## Lack of Available Services Supporting Recovery From SMI in the Western Cape

According to all participants, the public health services available for MHSUs tended to focus primarily on clinical recovery, irrespective of whether the services are offered at tertiary, secondary or primary levels of care. The participants highlighted that services within psychiatric hospitals were limited to medical stabilisation.

*So, psychiatric treatment does not think beyond the stabilisation, does not think how is this person going to get back into work and support themselves? So we [are] dealing with stabilisation, but the person does not live in a psychiatric hospital; they are only there for stabilisation. They have to go out into their family, community and work, but where is the further treatment and support? It is basically absent.* (OT3)

Service providers reported that the physical environment of the hospital prioritizes practical safety precautions to minimize the risk of bodily harm for the MHSUs over creating a healing space conducive to personal recovery:

*Often when you come into the environment, there is nothing. There is a table, and there is a chair, and they try to give the heaviest chair because they are assuming that the patient is going to pick up this chair and throw it at somebody. So, furniture everything is designed for safety, which is something big that you need to consider.* (OT3)

Tertiary hospitals also experience pressure for space when they have MHSUs needing urgent admissions while they cannot discharge others. As a result, service providers used the phrase “bed blockers” when referring to MHSUs stuck in the hospital system. The reasons for this varied:

*We were saying to our bosses [that] there is an elephant in this room, and the elephant is our bed blockers, our increasing burden, our patients staying longer in our hospital because families do not want them. There are no families. (P1)*

*What I have heard about Valkenberg is that substance use is a massive problem in terms of why people get admitted, drug-induced psychosis and things. I have heard in the past resentment from the staff in the wards. It is like they are taking bed space from people who is just an ordinary schizophrenic who needs that bed. There is some resentment. ‘It is your fault that you are here’ kind of stuff*. (SW3)

Participants indicate that the integration of mental health into primary levels of care “requires investment in mental healthcare at primary level, and there is no investment”. Once discharged, services available to MHSUs were reported to be primarily limited to collecting medication from a clinic at the primary healthcare level, where a lot of time is spent waiting to be attended to:

*The story of arriving there at 4 o’clock in the morning because otherwise, you are not going to be seen that day, I hear over and over again.* (D1)

Once discharged from the psychiatric hospital, those MHSUs with an SMI who are at risk of relapse are seen in their homes by the assertive community team (ACT). A participant who was previously on the ACT team shared, “the focus was trying to manage compliance to medication because obviously medication was a big part of why patients were relapsing”. Thus, according to participants, the focus of this service was to facilitate medication adherence.

*The main function was the home visit programme, so we went to the client’s home, assess the home circumstances and the families and then looked around medication compliance.* (OT1)

According to a few stakeholders, the non-profit sector offers personal recovery-focused services for “people who have been formally diagnosed with mental conditions like schizophrenia, depression, mood disorders”. Services provided by NPOs have a range of elements that service providers see as contributing towards personal recovery. One service provider shared their service package:

*Recovery is such a multi-faceted thing. Our services range from practical support, like clothing and food parcels and bus-fare through to life skills type projects, like cooking and projects that help with relaxation, leisure, stress management, perhaps art, and sport. Then we have our skills development side of things, and that again is a multifaceted space. They all have a thread running through them of the sort of basic things about hope for recovery, hope for something better, communication, social engagement and interaction, self-esteem, confidence building; there is a common thread.* (SW3)

## Facilitators and Barriers to Personal Recovery

The participants highlighted several facilitators and barriers to personal recovery. These factors varied and included: (i) service-related barriers; and (ii) difficulties with MHSUs community reintegration.

### Service-Related Barriers

To begin with, participants identified services’ focus on clinical recovery as a significant barrier to personal recovery. Service providers lamented that services lacked a “recovery-based focus”, and they noted a shortage of “intersectoral collaboration and partnerships” that could extend their work beyond clinical recovery. This is illustrated in the following quote:

*We only do the stabilisation and the other things; then outside of the multidisciplinary team is all the sectors- social services, education, labour. All those other departments speak to the recovery of the person with mental illness in the community [but] we have not got there yet.* (D2)

Second, the lack of consistent, long-term relational support offer to MHSUs by service providers acts as a barrier to personal recovery. According to service providers, support through long-term relationships between MHSUs and service providers was essential, especially for MHSUs living in supported living environments with limited family contact. In these environments, service providers can promote personal recovery through the therapeutic relationships they have with MHSUs:

*But it’s also because psychiatric patients, they want that. They want that reliable person. They want the consistency. They don’t want to have to repeat their story every time.* (N1)

Third, service providers shared that lack of flexibility and openness to MHSUs’ cultural beliefs can serve as a barrier to personal recovery. Providers described how “in Xhosa culture, if you get ill and if you do strange things and behaviours these symptoms could be directly correlated with schizophrenia, but it is viewed that the ancestors are calling you to become a sangoma” [traditional healer]. Service providers, therefore, need to discern between cultural practices, cultural beliefs and symptoms of mental illness. For example, one participant, an occupational therapist, shared:

*So, if the guy says ‘I am becoming a sangoma’, I will ask him: where are you going to practise and who are you going to practise on? If they can give me detail, then for me, there is a basis of truth. If there is no detail except, ‘I am going to kill everyone with my hands and heal everyone,’ then I am like, okay, delusion, right?* (OT1)

Consulting with colleagues was seen as an opportunity to expand service providers’ understanding of the MHSUs cultural context. Given the cultural nuances in the way symptoms are expressed and the cultural beliefs and understandings regarding the causes of these symptoms, many service providers highlighted the importance of consulting with colleagues when their backgrounds differed from that of the MHSUs they were seeing.

*Our Xhosa colleagues would say his family is very traditional, so it would not be outside the cultural norm to learn to become a sangoma. So, we have to see and do a little more investigation, and if it was appropriate, it isn’t a delusion.* (OT1)

### Difficulties with Community Reintegration

Service providers shared their perceptions of facilitators and barriers to personal recovery that MHSUs experienced once discharged from psychiatric services.

First, while the family was part of the MHSUs support network, service providers described how family dynamics and “toxic relationships that often exist” impacted on recovery and caused “an inevitable relapse” if not dealt with:

*And then, I met the family, and I realised the illness was not limited to the patient. So, people exist in systems and invariably, not invariably, but usually, the entire system is dysfunctional.* (D1)

*The families are not always supportive; they’re problematic families. So you don’t get the family that is supporting you in taking your medication or ensuring that you don’t go back to the substances or, you know, try and support recovery for that person.* (SW1)

Second, service providers highlighted unhealthy community environments as unconducive to personal recovery, stating that MHSUs “are going straight back to the community where all their problems started”, and there is a recognition that family holidays can also hinder recovery:

*Our patients will say to us: ‘I do not want to go home for Christmas because I know what is going to happen. Everyone is drinking or taking drugs. I will rather go* [home] *after Christmas’.* (SW3)

Third, socio-economic factors such as a lack of financial control, dependence on social grants and unemployment were perceived as some of the factors influencing MHSUs’ personal recovery. Service providers are mindful that MHSUs’ financial contributions from disability grants are essential to their household’s economic survival, and they ask themselves: “does the family depend on the grant?” as a determiner of whether the family wants the MHSU at home:

*I think for people who are fairly impoverished, the grant is quite an important source of income for families. But that is often the sole income in that house.* (SW4)

Service providers report on the dilemma faced by MHSUs when they need the income but are not allowed financial control over their social grant. For some MHSUs, not claiming eligibility for social assistance keeps the MHSUs and their family safe:

*He says: ‘if I get my disability grant, my family will take it away from me, and they will buy drugs with the money’. So he does not want a disability grant.* (V1*)*

Fourth, for those MHSUs not in receipt of a social grant, service providers reflected on the “struggle with unemployment” that MHSUs faced when they are on their personal recovery journeys and want to reintegrate back into their communities:

*So many of our clients get to a point where they are clean; they are doing what they need to do, but now what? Is this a worth it sort of thing? ‘So I am not employed, I do not have experience, no one wants to hire me, I am just sitting around doing nothing, and there is that lack of integration back into society and the community. And that is where we struggle the most, I think.* (D2)

Fifth, comorbid substance abuse was reported to impact personal recovery as it influenced how MHSUs managed themselves. For example, the comorbid diagnosis contributed to “the very high readmission rate because of the substances”. One participant indicated that “60% of our admissions” were due to substances, and relapses occurred “between six to 18 months” after discharge. One of the causes of this relapse was MHSUs inability to cope with difficulties they face and who then “self-medicate when they are getting unwell then they will use [substances] again”. This was reiterated in the following quote:

*They are self-medicating the feeling away. ‘So, tik makes me happy, weed makes me happy, and in a clinical perspective, I find that the instant gratification of that outweighs the consequences of coming back to the hospital and getting sick again.* (OT1)

Finally, service providers shared that the personal recovery of men is hindered by how they manage their problems or how society views their behaviours. Service providers suggest that “males take longer to present themselves, and that’s why they take longer to recover”. The personal recovery of men is also compromised by societal norms around masculinity and what it means to be a man, which says that “males are not supposed to have problems”. The stigma attached to having an SMI also impedes recovery as all behaviours displayed by male MHSUs are attributed to their SMI:

*Men do not access help. Men with mental illness very seldom go, ‘I need help’.They get drunk and beat someone up or get hooked on tik and becomes psychotic and so on.* (D1)

*It is expected for a female to have all the emotions and be hyper and feisty. But once you are a male with mental illness, you raise your voice, and the police will come and fetch you. And we have seen clients being readmitted with no psychotic features* (OT1*)*

## Discussion

This study resulted in several significant insights into how South African healthcare providers perceive personal recovery from SMI. First, results highlighted that although participants valued personal recovery, this is rarely addressed in available services which largely target clinical recovery. Second, personal recovery was viewed as a cyclical, complex process that takes place over time. Finally, barriers and facilitators to personal recovery from SMI in the South African context were highlighted.

To begin with, the findings echo the predominant focus on clinical recovery in healthcare settings in other parts of the world (Le Boutillier et al., 2015; Slade, 2009), including LMICs (Adewuya & Oguntade, 2007; Bila, 2019; Humphries et al., 2015; Rashed, 2015). Although service providers are aware of personal recovery, they focus on clinical recovery. Service providers manage competing demands concerning clinical versus personal recovery focused interventions. This is influenced by the priorities of their working environments, which favour a biomedical perspective because of resource constraints within the health system. In a systematic review exploring clinician and manager understanding of recovery-oriented mental health practice (Le Boutillier et al., 2015), service providers were seen as the health experts shaping their interventions and favouring a focus on stabilising or improving mental state through the use of medication. In LMICs, mental health service providers are in short supply and are predominantly based at specialised psychiatric facilities (Eaton et al., 2011). In South Africa, the public mental health budget makes up 5.0% of the public health budget and 86% of mental health care expenditure goes to inpatient care (Docrat et al., 2019; Petersen et al., 2019).

Despite efforts to integrate mental health into primary healthcare services in LMICs, like South Africa, progress remains slow due to existing infrastructure, budget and human resource shortages (Eaton et al., 2011). The limited resources allocated to primary levels of care results in a focus on managing SMI through psychotropic medication (Docrat et al., 2019). Although all the service providers in the study supported the South African Mental Health Policy and Strategic Plan 2013–2020 (Dept. of Health, 2014), that advocates for mental health to be integrated into primary healthcare, they reflected upon the lack of financial and human resources to offer these services. Challenges faced by mental health services in the primary care setting relate to inadequate infrastructure, poor organisation, shortage of medication, insufficient staff with mental health training, stigma towards people with SMI, long waiting times, lack of rehabilitation and mental health promotion services, limited psychosocial services and insufficient community-based services (Baker & Naidu, 2020; Sorsdahl et al., 2020; Wakida et al., 2018). Collaborative care models for common mental disorders have been explored to address these gaps, but the capability to implement these models varies considerably among primary care facilities and is driven mainly by attitudinal and resource differences across settings (Myers et al., 2019). Further, the application of these care models for the management of SMI needs to be investigated, especially in light of specialist resource shortages and the proposed benefits of utilising existing human resources within communities. Collaborative efforts across levels of care provide opportunities to transform mental health services and promote personal recovery. Collaboration with diverse stakeholders, including outside of the health sector (Fitts et al., 2020), emerged as a way to expand mental health services.

Service providers described personal recovery from SMI as a cyclical, complex process that takes place over time. These findings are consistent with definitions of personal recovery as described in previous research conducted in HICs and LMICs (Chen et al., 2018; Güner, 2014; Leamy et al., 2011; Llewellyn-Beardsley et al., 2019; Mathew et al., 2018; Nxumalo Ngubane et al., 2019; Rashed, 2015; Slade & Longden, 2015; Soygür et al., 2017; Subandi, 2015). Although service providers in this study supported personal recovery as a concept, they did not clarify how mental health services at primary and tertiary levels of care could support personal recovery in practice. Instead, they see clinical recovery as part of, and sometimes a pre-requisite for personal recovery. As a result, service providers prioritise clinical over personal recovery resulting in few resources being allocated to support personal recovery. An area of further consideration is the binary manner in which clinical and personal recovery is viewed. In South Africa, as in other LMIC countries, personal recovery starts once the MHSU is discharged back into their communities (Chen et al., 2018; Humphries et al., 2015; Nxumalo Ngubane et al., 2019; Rashed, 2015; Subandi, 2015), and not while they are in hospital.

Given the limited availability of personal recovery-focused services in the community, many people with an SMI experience multiple relapses. Service providers described high relapse rates amongst MHSUs and attribute relapses to crisis discharges, pressure for in-patient beds and non-adherence to medication (Botha et al., 2010). A recent study reported that 24% of mental health inpatients are readmitted within three months of a previous admission (Docrat et al., 2019). Frequent relapses are costly and result from various barriers MHSUs face, which impede their recovery. Some of the reasons for frequent readmissions have been cited in the literature. They include premature discharges, insufficient community resources, poor integration of mental health into primary healthcare, non-adherence to medication, homelessness, substance abuse, inadequate family support and diverse cultural responses to SMI (Botha et al., 2010; Habtamu et al., 2015; Jacobs & Coetzee, 2018; Lazarus, 2005; Smartt et al., 2019).

Service providers shared their views on some of the environmental and contextual barriers to personal recovery faced by MHSUs when returning to their communities. These included gender-based norms, stigma, family conflict, substance abuse, lack of long-term relational support, lack of money and unemployment. A scoping review on intersectional inequalities in mental health in HICs (Fagrell Trygg et al., 2019) included 20 studies that considered gender, socio-economic position, race, ethnicity, and sexual orientation. The findings suggest that intersectional inequalities in mental health across different population groups and settings hinder recovery. This is not surprising given the available literature indicating that social determinants of mental health play a role in personal recovery. In a systematic review of reviews conducted by Lund et al. (2018), social determinants of mental disorders were mapped according to the Sustainable Development Goals (SDGs) (Resolution, 2015). They included the following domains: demographic, economic, neighbourhood, environmental, social and cultural domains (Lund et al., 2018). The results indicated that coordinated intervention efforts between government services, civic societies and the private sector are needed when considering mental health system reforms. Given that personal recovery includes being able to participate in everyday life and everything that holds meaning and purpose for an individual, the factors influencing recovery extend past the confines of services offered by the health sector or the abilities of an individual with SMI. A scoping review on recovery from SMI in LMICs found that social networks and supportive relationships with others facilitated social inclusion and recovery (Gamieldien et al., 2021). If personal recovery happens in community and requires long term relational support, then service providers must consider where and how MHSUs can access this network once discharged from hospital.

## Implications for Future Research

This is one of the first studies in South Africa looking at personal recovery from SMI. Service providers recommend that diverse stakeholders and service sectors be involved in understanding and generating opportunities for personal recovery. MHSUs and their caregivers must be part of research initiatives as they can provide novel insights into personal recovery focused service priorities. Mental health reform must be cognisant of MHSUs social, economic, cultural and political environments as intersectional inequalities hinder personal recovery and community integration. Promoting personal recovery requires policies that advocate for and support the development of recovery based mental health servies across the continuum of care.

## Strengths and Limitations

A diverse range of mental health professionals were recruited for this study from the government-funded health services and the NPO sector. The study aimed to provide a detailed description of service providers understanding of the complex nature of recovery from SMI. The findings offer focused insights into service providers views on recovery along with possibilities for future research. We identify several limitations. First, although the sample included service providers from different disciplines, participants were likely not representative of mental health service providers in other sectors or other parts of South Africa. Second, the sample size was small. Third, we did not observe any practice and all findings were based on service provider reports. Fourth, the definition of recovery is contested, and the sample recruited might have had more knowledge on personal recovery than providers in other levels of health care.

## Conclusion

This study contributes to the growing interest in understanding service provider perspectives of personal recovery from SMI in LMICs. The findings highlight the distinction drawn by service providers regarding clinical and personal recovery. This distinction is primarily influenced by models of care and human resource constraints in mental health service delivery. While participants focused on clinical recovery, they acknowledged the importance of personal recovery, sharing their views on the multiple systemic challenges and contextual influences faced by MHSUs on their journeys towards personal recovery. Given the constraints in mental health service delivery, innovative interventions such as peer-support (Chien et al., 2019), collaborative care models (Petersen et al., 2019), task sharing (Brooke-Sumner et al., 2017; Hanlon, 2017), and telehealth (Medalia et al., 2020) offer opportunities to extend the reach of services.
